# Reward for a Cholera Specific

**Published:** 1849-12

**Authors:** 


*Reward for a cholera Specific.*—M. de Remmer, formerly a banker at Hamburg, and who died lately at Naples, has left, by will, a sum of 100,000 fr. to be given to any person who shall discover a remedy for cholera.

